# SSG-Net: A Multi-Branch Fault Diagnosis Method for Scroll Compressors Using Swin Transformer Sliding Window, Shallow ResNet, and Global Attention Mechanism (GAM)

**DOI:** 10.3390/s24196237

**Published:** 2024-09-26

**Authors:** Zhiwei Xu, Tao Liu, Zezhou Xia, Yanan Fan, Min Yan, Xu Dang

**Affiliations:** School of Mechanical and Electrical Engineering, Lanzhou University of Technology, Lanzhou 730070, China; 231080203005@lut.edu.cn (Z.X.); 222080201056@lut.edu.cn (Z.X.); 222085501022@lut.edu.cn (Y.F.); 211080203004@lut.edu.cn (M.Y.); 221080201005@lut.edu.cn (X.D.)

**Keywords:** scroll compressor, global attention mechanism, fault diagnosis, deep learning

## Abstract

The reliable operation of scroll compressors is crucial for the efficiency of rotating machinery and refrigeration systems. To address the need for efficient and accurate fault diagnosis in scroll compressor technology under varying operating states, diverse failure modes, and different operating conditions, a multi-branch convolutional neural network fault diagnosis method (SSG-Net) has been developed. This method is based on the Swin Transformer, the Global Attention Mechanism (GAM), and the ResNet architecture. Initially, the one-dimensional time-series signal is converted into a two-dimensional image using the Short-Time Fourier Transform, thereby enriching the feature set for deep learning analysis. Subsequently, the method integrates the window attention mechanism of the Swin Transformer, the 2D convolution of GAM attention, and the shallow ResNet’s two-dimensional convolution feature extraction branch network. This integration further optimizes the feature extraction process, enhancing the accuracy of fault feature recognition and sensitivity to data variability. Consequently, by combining the global and local features extracted from these three branch networks, the model significantly improves feature representation capability and robustness. Finally, experimental results on scroll compressor datasets and the CWRU dataset demonstrate diagnostic accuracies of 97.44% and 99.78%, respectively. These results surpass existing comparative models and confirm the model’s superior recognition precision and rapid convergence capabilities in complex fault environments.

## 1. Introduction

Scroll compressors, recognized for their environmental friendliness, high efficiency, and energy conservation, are extensively utilized in critical sectors such as food processing, refrigeration, and transportation [1,2,3]. These devices are essential for maintaining system reliability and operational efficiency, directly impacting the overall performance of the machinery [4,5]. High-end scroll compressors are typically subjected to rigorous conditions, operating at high speeds and pressures, which expose them to an increased risk of faults [6]. Such malfunctions can not only disrupt operations but may also lead to catastrophic failures [7]. Consequently, precise and effective fault diagnosis in scroll compressors is crucial for ensuring industrial safety and functionality.

In recent years, fault diagnosis methods have been categorized into three main types: model-based detection methods [8], diagnostics based on expert knowledge [9], and data-driven approaches [10]. While model-based detection methods and expert knowledge-based diagnostics were extensively applied in the past, they have demonstrated significant limitations in handling complex industrial environments. These approaches are heavily dependent on accurate mechanistic models and expert insights, which pose challenges when adapting to complex and nonlinear fault conditions. Furthermore, the maintenance of such models entails substantial costs. The limitations of model-based and expert knowledge-based diagnostics are primarily due to the difficulties in constructing precise mechanistic models, their lack of adaptive learning capabilities, and the associated high maintenance requirements [11]. In contrast, data-driven methods, which do not rely on specific mechanistic models or expert knowledge, have gained prominence. The rapid development of modern machinery, sensor technology, and the Industrial Internet of Things (IIoT) has facilitated real-time data collection and storage from rotating machinery, significantly enhancing the potential of these methods for big data utilization in fault prediction and analysis [12]. Traditional machine learning algorithms such as support vector machines [13,14], extreme learning machines [15,16], and K-means [17,18,19] have been widely applied in fault diagnostics with considerable success. However, despite these achievements, such methods heavily depend on manual feature extraction, which limits their ability to fully capture the complex features present in fault data. Moreover, the shallow network structures of these algorithms make them less effective in processing high-dimensional and complex nonlinear data. For instance, Fu et al. [20] introduced an ensemble empirical mode decomposition (EEMD)-based diagnostic method capable of effectively diagnosing faults under stochastic noise, noted for its speed, low error rate, and stable performance. However, due to the high number of iterations and slow decomposition speed of EEMD, Sun et al. [21] proposed a fault feature extraction method that combines empirical mode decomposition (EMD) with an improved Chebyshev distance, which has been validated experimentally. Chen et al. [22] proposed an intelligent fault diagnosis model based on a multi-core support vector machine (MSVM) optimized using a chaotic particle swarm optimization (CPSO) algorithm. This model demonstrated good generalization capability and diagnostic accuracy. Similarly, Ma et al. [23] introduced a fault classification and diagnosis method based on a BP neural network ensemble. In this method, multiple sub-BP networks were employed for separate diagnoses, which increased the accuracy of fault diagnosis from 95% to 99.5%. However, due to the shallow network structure and the insufficient feature extraction capability of traditional machine learning algorithms, deeper micro-features contained in fault data could not be explored and extracted [24]. Consequently, this limitation hindered the further improvement of diagnostic accuracy.

Compared to traditional machine learning methods, modern fault diagnosis methods based on deep learning offer the advantage of eliminating the need for manual feature extraction. However, despite the significant progress made by deep learning in automating feature extraction, current models still face limitations in their ability to fully capture comprehensive features. Specifically, methods such as Convolutional Neural Networks (CNNs) excel in extracting local features but struggle to effectively capture global signal dependencies, particularly in complex fault data, where deeper global information may be overlooked. Moreover, these methods autonomously extract valuable information from raw data and subsequently perform classification tasks [25]. Notably, significant progress has been made in intelligent fault diagnosis using convolutional neural networks (CNNs) [26]. For example, Dong et al. proposed a CNN model with a compound attention mechanism for identifying faults in supersonic aircraft [27]. Additionally, Zhao et al. designed an adaptive intra- and inter-class CNN model that effectively identifies gear faults even under varying speed conditions [28]. However, CNN models primarily focus on capturing local features in signals. Consequently, they may face challenges in establishing global dependencies, which limits their ability to effectively extract fault information and achieve robust generalization performance, especially when dealing with small sample sizes [29]. He et al. [30] proposed a feature-enhanced continuous learning method that allows a diagnostic model to continuously and adaptively acquire knowledge of new fault types, effectively mitigating the forgetting of features and enhancing the model’s fault detection capabilities. Similarly, Seimert et al. [31] developed a diagnostic system based on a Bayesian classifier capable of classifying and diagnosing healthy and damaged bearings. In addressing unknown fault types that may arise in practical applications, Yang et al. [32] proposed a multi-head deep neural network (DNN) based on a sparse autoencoder. This network learns shared encoded representations for unsupervised reconstruction and supervised classification of monitoring data, enabling the diagnosis of known defects and the detection of unknown ones. Furthermore, Lei et al. [33] introduced an intelligent fault diagnosis method based on statistical analysis, an improved distance evaluation technique, and an adaptive neuro-fuzzy inference system (ANFIS) to identify different fault categories and severities. Liu et al. [34] proposed a new algorithm—the extended autocorrelation function—to capture weak pulse signals in rotating machinery at low signal-to-noise ratios. They further proposed an improved symplectic geometric reconstruction data augmentation method, which offers higher accuracy and convergence capabilities in the fault diagnosis of imbalanced hydraulic pump data [35]. Moreover, Deng et al. [36] have developed an end-to-end time series forecasting method known as D-former for predicting the Remaining Useful Life (RUL) of rolling bearings. This method is specifically designed to extract degradation features directly from the original signal. In addition, Jin et al. [37] introduced a novel fault diagnosis method named the multi-layer adaptive convolutional neural network (MACNN) to tackle the challenge posed by deep learning models’ heavy reliance on consistent feature distribution across training and testing datasets. The MACNN utilizes multi-scale convolution modules to extract low-frequency features and effectively address classification problems.

The development of automated fault identification models typically relies on large-scale standardized datasets. However, in practical applications, acquiring well-labeled fault data is both time-consuming and labor-intensive, often leading to model overfitting due to the scarcity of fault data. To address this issue, data augmentation techniques have emerged as an effective solution. For instance, Taylor and Nitschke [38] significantly improved the detection performance of CNNs. Similarly, Fernandez et al. [39] increased the number of minority class samples by creating synthetic samples between existing samples of the minority class, ensuring that the model does not overlook these minority samples during training. Furthermore, He et al. [40] enhanced the number of minority class samples using adaptive synthetic techniques, thereby improving the model’s focus and classification performance on difficult-to-classify data points and enhancing overall model performance and accuracy. A self-paced fault adaptive diagnosis (SFAD) method based on a self-training mechanism and target prediction matrix constraint was proposed by Jiao et al. [41], achieving model adaptation using only unlabelled target data. Additionally, Hu et al. [42] employed relocation techniques to simulate data under different rotational speeds and workloads, effectively increasing the sample size. Moreover, Yang et al. [43] proposed a diagnostic model using a polynomial kernel-induced MMD (PK-MMD) distance metric to identify the health status of locomotive bearings. However, traditional data augmentation methods often lead to model overfitting and fail to address data distribution edge cases. To overcome these limitations, a data augmentation method based on generative adversarial networks (GANs) has been proposed [44]. This method leverages the capabilities of GANs to generate new, synthetic samples that exhibit high variability while maintaining statistical similarity to real data. This approach effectively increases data volume, particularly in cases of minority class data scarcity, avoiding the overfitting issues associated with simple sample repetition and better exploring the edge regions of data distribution, thereby improving the model’s generalization to uncommon scenarios. Jiao et al. [45] proposed a self-training reinforced adversarial adaptation (SRAA) diagnostic method based on a dual classifier difference metric. Similarly, Zhao et al. [46] introduced a deep residual shrinkage network to enhance the feature learning capability of high-noise vibration signals, achieving high fault diagnosis accuracy. Additionally, Zhang et al. [47] utilized multiple learning modules and a gradient penalty mechanism to significantly improve the stability of the generative model and the quality of generated data. Despite the demonstrated potential of GANs in bearing fault diagnosis, their limitations cannot be overlooked. Firstly, current GAN-based research mainly focuses on the processing of one-dimensional signals, which may neglect the challenges of extracting complex and deep features from one-dimensional data, potentially leading to the loss of critical information and affecting the quality of generated data. Secondly, the training process of GANs frequently encounters the issue of gradient vanishing. Although Wasserstein GAN (WGAN) [48] alleviates this problem by introducing a Lipschitz constraint, its capability to fit complex data samples remains limited, impacting model efficacy and potentially leading to the waste of computational resources. In recent years, machine learning and deep learning methods have made significant progress in the field of fault detection, with CNNs and Recurrent Neural Networks (RNNs) excelling in feature extraction and pattern recognition. However, these methods have limitations in dealing with global features and long-range dependencies. For this reason, Transformer models have become an important tool in fields such as computer vision and natural language processing due to their powerful global feature extraction capabilities [49,50]. Moreover, the application of the Transformer model in mechanical equipment fault identification has become increasingly widespread, and some scholars have utilized it for modeling and detecting mechanical equipment faults [51]. For instance, Tang et al. developed a Transformer model specifically designed for bearing fault identification under variable conditions [52], while Ding et al. introduced a time-frequency analysis-integrated Transformer for detecting faults in rotating machinery [53]. Furthermore, Zhao et al. proposed an Adaptive Threshold and Coordinate Attention Tree Neural Network (ATCATN) for hierarchical health monitoring, which accurately identifies the location and size of faults in aircraft engine bearings, even under severe noise interference [54]. Despite their excellence in capturing global features, the performance of Transformer models heavily relies on large datasets and consumes substantial computational resources. The technical challenges primarily include: firstly, the multi-head self-attention mechanism requires extensive matrix multiplications during construction, significantly increasing the demand for computational resources; secondly, traditional convolution kernels used for feature extraction also entail high computational costs; lastly, although feature dimension reduction achieved through pooling layers aids in model light-weighting, it often sacrifices detailed data features. Therefore, developing an efficient and lightweight self-attention mechanism and convolution operations becomes crucial. Such methods not only reduce reliance on computational resources but also effectively downsample features while retaining critical information, providing optimal model deployment solutions for resource-constrained environments.

Fault diagnosis for scroll compressors is still in the early stages of development, with current technologies struggling to meet the demands for both efficiency and accuracy, particularly in complex operational environments. Existing methods predominantly rely on traditional feature engineering, which faces significant challenges in capturing the intricate and often subtle fault patterns present in scroll compressors. To address these challenges, this study introduces an innovative fault diagnosis method based on an enhanced design of two-dimensional convolutional neural networks (2D CNNs). In this preliminary research, experiments were conducted under ideal conditions to validate the method’s effectiveness. The results demonstrate improved diagnostic accuracy and robustness, providing a solid foundation for future studies. These future investigations will explore the method’s performance in more complex and challenging conditions. The key innovations of this approach include:

(1) Multiscale Feature Extraction: The application of the Short-Time Fourier Transform (STFT) converts one-dimensional time-series signals into two-dimensional images. This transformation allows the model to perform feature extraction at multiple scales, thereby effectively capturing minor variations in the signal. By utilizing the extensive spectral information contained in two-dimensional images, the model achieves a comprehensive understanding and analysis of mechanical equipment operation. Consequently, this leads to higher fault detection rates in practical applications and ensures the accuracy and reliability of diagnostic results.
(2) Hybrid Architectural Design: The integration of Swin Transformer’s window attention mechanism, the global attention mechanism of the Global Attention Mechanism (GAM) Attention, and the shallow 2D convolution feature extraction branch network of ResNet, has been shown to significantly enhance the model’s generalization ability and sensitivity to data. Moreover, this hybrid architecture optimizes the feature extraction process, thereby improving the model’s stability and accuracy in handling complex data. Additionally, it minimizes computational resources, thus increasing the model’s adaptability and performance in diverse data environments.
(3) Deep Feature Fusion: The model integrates global spatial and local features extracted by various branch networks using pooling technology. This multilevel feature fusion enables the model to more effectively integrate and express information from different data scales, thereby greatly enhancing its expressiveness and robustness. As a result, the application of deep feature fusion allows the model to exhibit higher adaptability and diagnostic precision when confronted with complex and variable fault signals, significantly improving the reliability and efficiency of fault diagnosis.

## 2. Preliminaries

### 2.1. GAM Module

CNNs have been extensively applied across various fields, particularly in computer vision, and are highly regarded by researchers for their exceptional performance. Moreover, convolutional attention mechanisms have provided CNNs with a straightforward and efficient method for feedforward attention processing. Considerable research has been conducted on the performance improvements brought by attention mechanisms in image classification tasks. However, some challenges remain.

**Figure 1:**
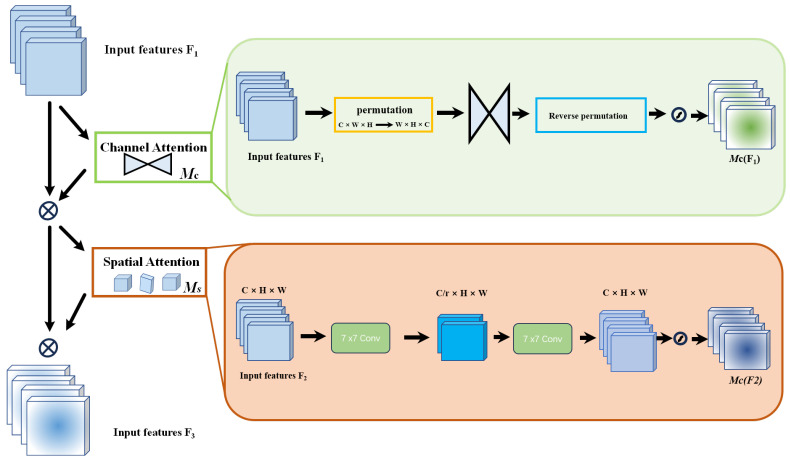
GAM module structure diagram.

For instance, the SENet [55] model may suffer from inefficiencies when suppressing features unrelated to the target task, consuming significant resources or time for pixel-level processing operations, which impacts the overall efficiency of model training. To address this, the CBAM [56] model implements both spatial and channel attention mechanisms. The channel attention mechanism compresses the feature map along the spatial dimensions, while the spatial attention mechanism compresses along the channel dimensions, producing new features. Conversely, the BAM [57] model operates two attention mechanisms in parallel but overlooks the interaction between channel and spatial attention. The GAM [58] model, on the other hand, enhances the interactions across spatial and channel dimensions, enabling the capture of critical feature information across three dimensions. Figure 1 illustrates the structure of the GAM module.

The GAM is designed to enhance neural network performance by integrating sophisticated channel and spatial attention mechanisms. Specifically, the channel attention component is implemented using a multi-layer perceptron (MLP) with two hidden layers and an activation function to introduce non-linearity. Dimensionality is effectively reduced through linear transformations, nonlinear enhancements are applied, and features are remapped to their original dimensions to isolate key channel-specific information. Complementary spatial attention is achieved through two convolutional layers and batch normalization, which accentuate spatial details and stabilize them. This process culminates in a synthesized feature profile that bolsters the model’s contextual understanding. This dual mechanism not only emphasizes relevant features at different scales and locations but also integrates these features into a cohesive, attention-weighted representation through a sophisticated fusion process. Consequently, essential global features are captured, significantly improving the model’s efficiency and accuracy in complex tasks such as classification and detection. Therefore, overall neural network performance is enhanced in demanding analytical environments.

### 2.2. ResNet Model

As one of the most iconic architectures in the field of convolutional neural networks, ResNet [59] is highly esteemed by the academic community for its innovative introduction of residual structures, which have significantly enhanced network performance. The residual structure addresses the problem of gradient explosion that arises as neural networks deepen. In a residual module, the input is directly added to the output of the module via identity mapping, ensuring that each layer feeds into the next with minimal loss of information. Consequently, this design allows deeper residual networks to consist of multiple such residual modules. Each module effectively fits the errors of its preceding classifier, thereby improving classification capabilities. The structure of a residual module is defined as follows:

(1) $$y=F\left(x,\;\left\{W_{i}\right\}\right)+x$$

In the formula, x and y represent the input and output vectors of the residual module, respectively. The term F(x, {Wi}) denotes the trainable residual mapping. For Equation (1) to hold, the dimensions of x and F(x, {Wi}) must coincide. If dimensionality is altered due to pooling, it becomes necessary to incorporate an identity mapping within the shortcut connection to ensure dimensional consistency. The corresponding formula is described as follows:

(2) $$y=F\left(x,\;\left\{W_{i}\right\}\right){+W}_{s}x$$

**Figure 2:**
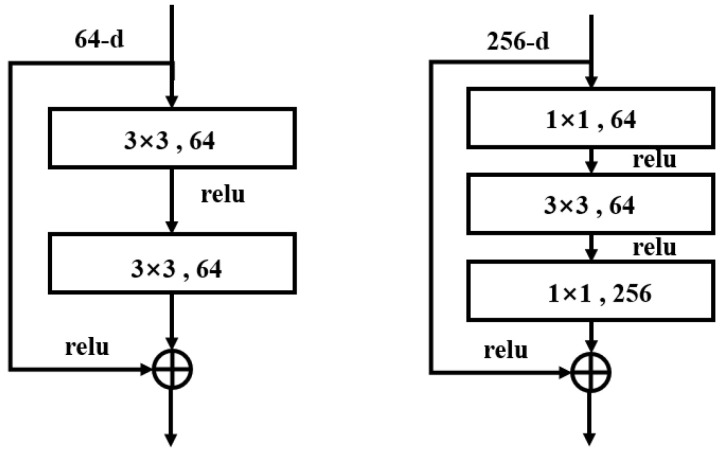
Two different residual structure diagrams.

The basic structure of the residual module is shown in Figure 2. The residual module employs a bottleneck block structure, utilizing 1 × 1 convolutional kernels to reduce the dimensionality of the feature maps, followed by another 1 × 1 convolution to restore the dimensions. This design reduces computational complexity while maintaining network depth, thereby enhancing model efficiency. Alternatively, a basic block structure, consisting of two 3 × 3 convolutional layers, is used. This basic block structure is typically employed in shallower ResNet models, where continuous 3 × 3 convolutions extract features while preserving the dimensions of the feature maps. Overall, the ResNet architecture fundamentally enhances traditional CNNs by adding a residual layer, significantly boosting network performance while maintaining very low complexity.

### 2.3. Swin Transformer Model

The Swin Transformer model, an innovative neural network architecture introduced by Google in 2017, relies solely on the attention mechanism, discarding traditional recurrent and convolutional network structures. This architecture has been extensively applied in various artificial intelligence scenarios, including machine translation, object detection, and audio processing. By utilizing a fully attention-based design, the Swin Transformer model enables parallel processing of input data, significantly accelerating training speed. Its multi-head attention mechanism allows the model to concurrently focus on multiple positions within a sequence, thereby enhancing its comprehension of complex contexts. Additionally, the Swin Transformer’s structure is highly scalable, allowing for the addition of more layers or heads to tackle more complex tasks, thus excelling in the processing of large-scale datasets. Furthermore, long-range dependencies are captured more effectively, making the Swin Transformer suitable for various languages and tasks. From text processing to computer vision, the Swin Transformer demonstrates exceptional adaptability and broad application potential.

**Figure 3:**
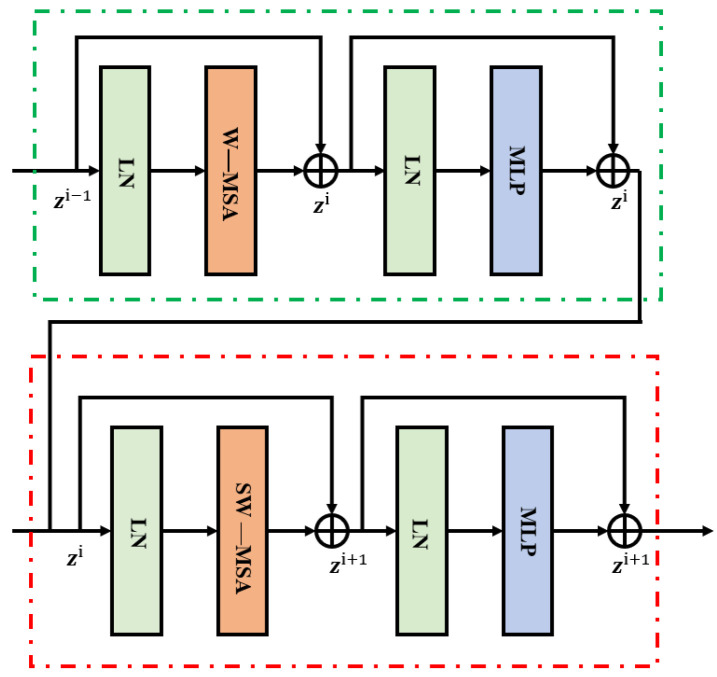
Swin Transformer architecture diagram.

In Figure 3, the key components of the Swin Transformer model are clearly illustrated, including Windowed Multi-Head Self-Attention (W-MSA), Shifted Windowed Multi-Head Self-Attention (SW-MSA), MLP, and Layer Normalization (LN). The input part of the model consists of a positional encoding layer and an embedding layer, while the output part mainly includes a linear layer and a SoftMax layer. At the core of the model, both the encoder and decoder are comprised of N layers, each consisting of multi-head attention layers, feedforward fully connected layers, normalization layers, and residual connection layers. The multi-head attention layers are composed of multiple attention heads, which help prevent the model from overfitting.

The core feature of the Swin Transformer is its self-attention mechanism, which transforms outputs by leveraging the internal correlations of data sequences. This mechanism involves Queries, Keys, and Values, each represented as vectors. The Query identifies key information within the input data, the Key represents the basic information of the data, and the Value contains specific data corresponding to the Key. The self-attention mechanism calculates and scales the dot product of feature vectors to analyze their similarity. This process addresses long-range dependencies and efficiently highlights important information while reducing reliance on external information. By emphasizing crucial information and ignoring irrelevant content, the goal of reducing dependence on external information is achieved. The corresponding computations can be represented as follows, where W^q^, W^k^, and W^v^ are the weight matrices for Queries, Keys, and Values, respectively:

(3) $$Q={\mathrm{XW}}^{q}$$

(4) $$K={\mathrm{XW}}^{K}$$

(5) $$V={\mathrm{XW}}^{v}$$

(6) $$\mathrm{Attention}\left(Q,\;K,\;V\right)=\mathrm{Softmax}\left(\frac{{\mathrm{QK}}^{T}}{\sqrt{\dim}}\right)V$$

In the Swin Transformer model, the Query (Q), Key (K), and Value (V) matrices are generated by multiplying the input data X with the corresponding weight matrices W^q^, W^k^, and W^v^. Here, dim represents the dimensionality of the Q, K, and V matrices and is used to scale the calculations, ensuring stability. The self-attention mechanism enables the model to focus on the important information within the input features. However, a single self-attention mechanism can only learn information within a single informational space. To more comprehensively understand the key information in the input sequence, the Swin Transformer model introduces the multi-head attention mechanism. This mechanism employs multiple self-attention layers in parallel, allowing the model to extract and focus on critical features from different informational spaces simultaneously, thereby obtaining richer and more integrated feature representations.

The specific implementation process is as follows: the input is divided into multiple parts, each part is processed separately using different weight matrices, and then these results are combined to form the final output. This approach enhances the model’s ability to understand the input data from multiple dimensions. The multi-head attention mechanism is computed as follows:

(7) $$\mathrm{MultiHead}\left(Q,K,V\right)=\mathrm{Concat}\left(h_{1}{,h}_{2}{,\ldots ,h}_{n}\right)\cdot W$$

(8) $$h_{i}=\mathrm{Attention}\left({\mathrm{Xw}}_{h}^{q}{,\;\mathrm{Xw}}_{h}^{k}{,\;\mathrm{Xw}}_{h}^{v}\right),\;h=1,2,\ldots ,n$$

In the multi-head attention mechanism, the input X is multiplied by the weight matrices W^q^, W^k^, and W^v^ to generate the Query Q, Key K, and Value V matrices for each attention head. Here, n represents the total number of attention heads. The results of each head, hi, are computed and then combined through concatenation (Concat) to form the final output. This network structure is designed to process multiple informational dimensions in parallel, thereby enhancing the model’s representational capacity and information capture efficiency. The network structure is illustrated as follows.

## 3. Network Structure

Based on the foundational theories discussed in Section 2, the complete details of the compressor fault diagnosis method utilizing the Swin Transform–Shallow Resnet—GAM (SSG-Net) will be comprehensively described in the following section.

### 3.1. Construction of the SSG-Net Model

**Figure 4:**
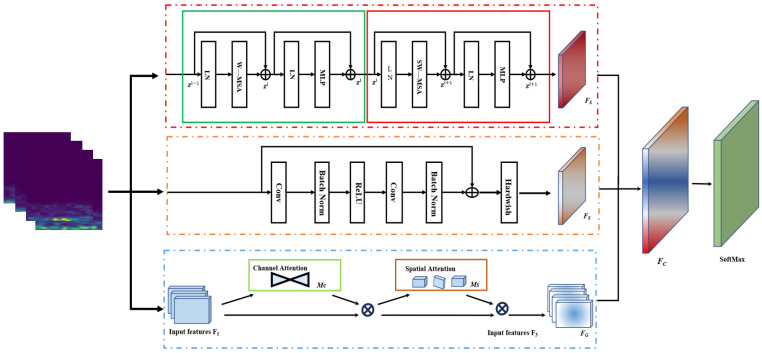
SSG-Net model structure.

This paper presents a multi-channel global attention mechanism-based model for compressor fault detection. Figure 4 illustrates the architecture of the proposed multi-channel SSG-Net model, which consists of three channels, each dedicated to extracting distinct feature information.

First, the initial channel employs a sliding window attention mechanism based on the Swin Transformer to extract local features from fault images. By calculating attention within each sliding window, this mechanism effectively captures relationships between features. Moreover, the sliding window approach allows the model to process each region of the image and extract local features. Additionally, the overlap between adjacent windows ensures continuity of local information, enabling the model to capture feature dependencies in the time-frequency diagram more accurately and extract critical global information from the signal. However, this method faces certain limitations in extracting fine-grained temporal details from shallow features.

Next, the second channel utilizes a shallow ResNet as the backbone network for feature extraction. The residual structure enables the model to build deeper networks, which enhances its feature extraction capabilities. Additionally, the alternating use of convolutional layers and batch normalization layers ensures stability during training, leading to the effective extraction of high-level feature representations. By retaining more spatial details and low-level features, the model is provided with finer-grained input features, thus improving the overall feature representation capability.

Finally, the third channel employs a CNN2d convolutional pooling network, which is based on the global attention mechanism (GAM-Attention). GAM-Attention computes attention weights across the entire image, prioritizing the key features of fault images. Within this network, the combination of channel attention and spatial attention enables the model to focus on essential features and capture global relationships. Consequently, this feature enhancement method significantly improves the model’s fault detection capabilities.

In summary, the multi-channel design of the SSG-Net model, which integrates sliding window attention, global attention mechanisms, and residual networks, offers a comprehensive approach to capturing both local and global features. This design effectively provides robust fault detection for compressors.

Finally, the SSG-Net model integrates features from three channels, encompassing local, global, and multi-scale information. This comprehensive integration allows the model to effectively utilize diverse features, facilitating the recognition of both global and local details and thereby enhancing fault detection accuracy. The model amalgamates the feature outputs from each module, incorporating long-range dependencies, channel attention, spatial attention, and local feature extraction. This fusion process ensures that the model leverages all available information. Consequently, the model can capture the global features of the entire image without neglecting local details. Ultimately, the combined features are input into a SoftMax classifier for fault classification.

### 3.2. SSG-Net Detection Framework

The core steps of the SSG-Net model consist of raw data preprocessing, feature extraction, and classification. Raw data alone typically do not provide sufficient information for effective feature extraction and meeting the requirements of deep learning models. Specifically, one-dimensional vibration signals lack explicit frequency information, despite offering amplitude information over time. Therefore, a Short-Time Fourier Transform (STFT) is employed to convert these signals into two-dimensional time-frequency images. This transformation enables a clear representation of the signal’s spectral information as it varies over time, significantly enriching the data available for subsequent feature extraction.

The preprocessing step begins with the application of STFT to the one-dimensional vibration signals. The STFT decomposes the signal into its constituent frequencies over short time windows, producing a time-frequency representation. This representation captures how the signal’s frequency content changes over time, providing a detailed view of both transient and steady-state components. The resulting two-dimensional images, known as spectrograms, contain rich information crucial for accurate fault detection.

In the feature extraction phase, traditional single-channel models may encounter significant challenges when learning from long-sequence data. These models often struggle to fully integrate local and global features, potentially leading to information loss and reduced prediction accuracy. To overcome these limitations, a novel architecture with multiple feature extraction pathways has been introduced. This model comprises three distinct branch networks, each designed to capture different aspects of the data. First, the Local Feature Extraction Branch focuses on fine-grained local details within the time-frequency images, using convolutional layers with small receptive fields to capture subtle variations and transient features. Second, the Multi-Scale Feature Extraction Branch employs a range of receptive fields to capture features at various scales, ensuring a comprehensive representation that includes both fine and coarse details. Finally, the Global Feature Extraction Branch aims to capture long-range dependencies and broader contextual information by utilizing larger receptive fields. These three branches work in parallel to extract a rich set of features from the input data, which are then passed through attention mechanisms, including channel and spatial attention, to enhance the most relevant aspects while suppressing noise and redundancy.

**Figure 5:**
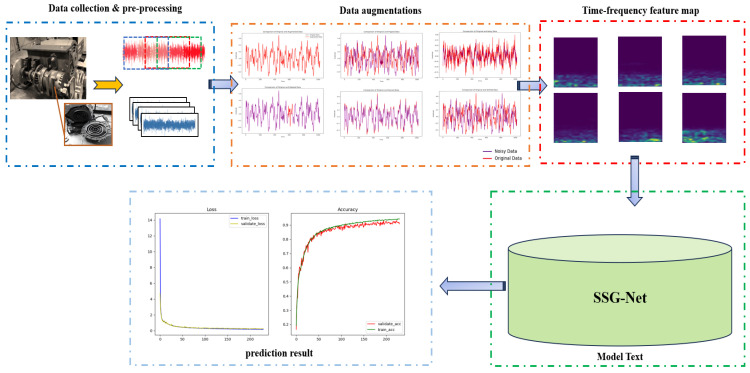
SSG-Net framework flowchart.

Furthermore, a C-pooling fusion technique is applied to compressor fault diagnosis, ensuring a more effective solution for capturing comprehensive feature information. The specific structure of this model is depicted in Figure 5.

### 3.3. Data Preprocessing

Data augmentation plays a crucial role in generating different views of positive and negative sample pairs, enabling robust representations to be learned by the model. The experiments demonstrate that appropriate data augmentation can enhance the model’s ability to learn from a limited amount of data. Although data augmentation methods are well-established in computer vision, they are less common in vibration signal analysis.

To expand the training data and improve the model’s robustness, several time-domain signal augmentation methods were introduced. These include masking, gain adjustment, shifting, fading, fade-out, horizontal flipping, and vertical flipping. Masking selectively applies processing effects to certain areas of the signal, simulating missing or corrupted data. Gain adjustment modifies the signal’s amplitude, enhancing its brightness or contrast. Shifting moves the signal forward or backward in time, creating temporal variations. Fading logarithmically increases or decreases the amplitude over a random interval, while fade-out gradually weakens or strengthens the signal at random points. Horizontal flipping reverses the time sequence of the signal, and vertical flipping inverts the amplitude values, altering the signal’s phase. These augmentation techniques collectively enhance the diversity and representativeness of the training data, leading to more robust feature learning and improved fault diagnosis performance.

**Figure 6:**
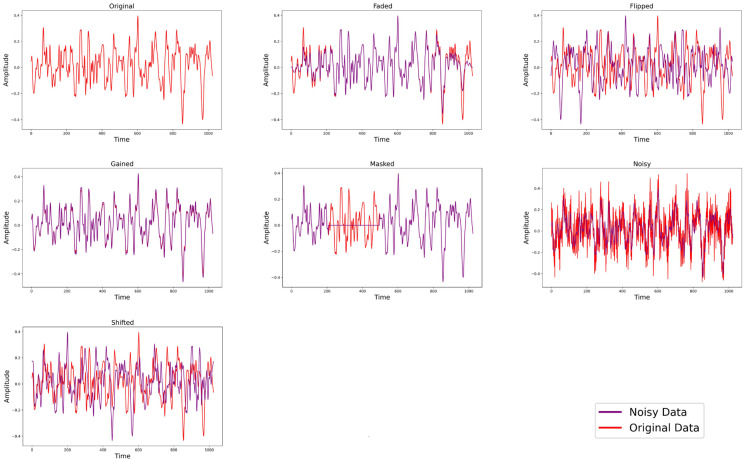
Visualization of fault signal augmentation methods.

Figure 6 illustrates the effects of these signal augmentation methods on the experimental dataset. In each training batch, six augmentation methods were selected and applied to the time-series data. Notably, even though multiple versions of the time-series data were generated, the original batch size was maintained, ensuring data consistency and comparability. This approach not only effectively enhances the model’s performance but also increases the scientific validity of the experiment.

**Figure 7:**
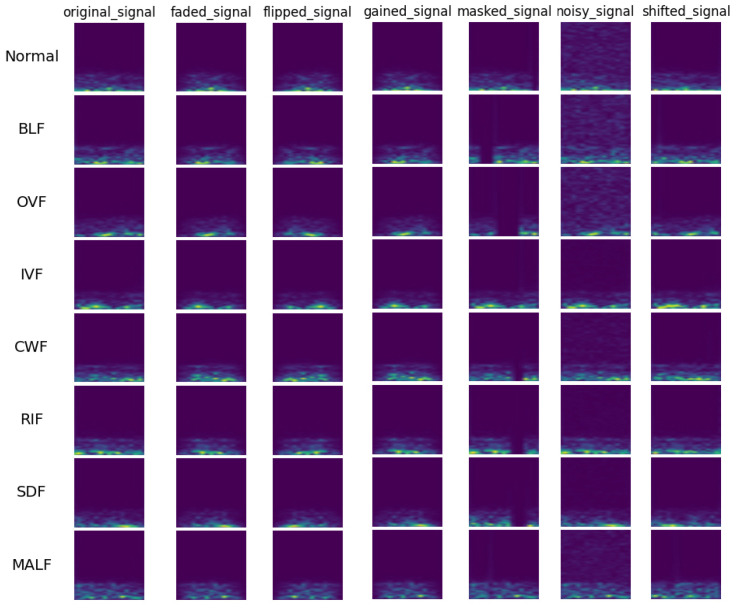
Visualization of STFT examples for augmented fault signals.

The output spectrum of the Short-Time Fourier Transform (STFT) can be represented as a two-dimensional matrix, with time along the horizontal axis and frequency along the vertical axis. The matrix elements denote the signal’s power or amplitude at the corresponding time and frequency. This two-dimensional image, known as a spectrogram, clearly displays the evolution of the signal over time and frequency. By transforming one-dimensional time-series signals into two-dimensional time-frequency images using STFT, the model can better understand and analyze both the frequency and time-domain characteristics of the signals. This transformation provides rich information for subsequent detection tasks. As depicted in Figure 7, the time-frequency spectrogram obtained through STFT serves as the input for model training.

## 4. Experimental Results and Analysis

In this section, the predictive performance of the proposed SSG-Net method is evaluated through two experimental case studies. Furthermore, the results are compared with several mainstream data-driven methods to validate the superiority of the proposed approach in compressor fault diagnosis.

### 4.1. Scroll Compressor Dataset

**Figure 8:**
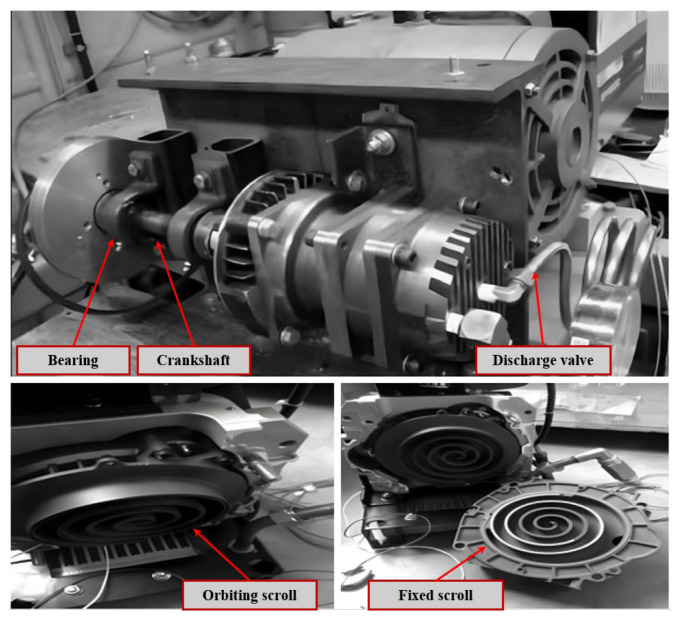
Scroll compressor experimental prototype.

The experimental data utilized in this paper originate from the scroll compressor prototype of the Lanzhou University of Science and Technology Scroll Mechanical Research Institute (Lanzhou, China), as depicted in Figure 8. The prototype operates at a speed of 3120 r/min, a frequency of 52.1 Hz, and a sampling frequency of 2000 Hz. The equipment used includes YD-5-2 and YD-8 piezoelectric sensors (Far East Vibration Measurement (Beijing) System Engineering Technology Co., Ltd., Beijing, China), a PCI-6221 data acquisition device (National Instruments, Austin, TX, USA), and an FRN55P11S-4CX type inverter (Fuji Electric, Tokyo, Japan). The sensors were installed at various points: the top of the scroll compressor, the engagement place of the static and dynamic scroll disks, the motor mounting position, and the bottom of the scroll compressor, corresponding to the external and internal mechanisms of the scroll compressor. Consequently, a total of seven types of fault signals and normal operation signals were collected. These fault signals include bearing loosening faults (BLF), inlet valve faults (IVF), outlet valve faults (OVF), rotor imbalance faults (RIF), crankshaft wear faults (CWF), scroll disk faults (SDF), and mechanical assembly loosening faults (MALF).

In this experiment, the PyTorch 1.9.1 deep learning framework was utilized on a hardware platform comprising an AMD Ryzen 5800H CPU (16 GB RAM) and an NVIDIA GeForce RTX 3060 GPU (6 GB VRAM) running on the Windows 11 operating system. Additionally, CUDA 11.1, CuDNN 8.2.1 (compatible with cu111), OpenCV 4.9.0.80, and related libraries were employed to train and test the compressor fault detection model.

In this experiment, the dataset was divided into three subsets: the training set, the test set, and the validation set. As shown in Table 1, each subset was labeled from 0 to 7, representing different types of signals. Specifically, each type included 1575 training samples, 450 test samples, and 225 validation samples. These samples were generated using an overlapping sampling method, with each sample having a length of 5120 and an offset of 2560.

In this study, ablation experiments involving various activation functions were first conducted to identify the most appropriate activation function for the SSG-Net model. The experimental results revealed that the SSG-Net model achieved optimal performance with the Hardswish activation function, attaining an accuracy of 97.44% and a loss value of 0.074. Table 2 provides a summary of the specific performance metrics of the SSG-Net model when different activation functions were used. The Hardswish activation function combines smoothness with nonlinearity, effectively mitigating the vanishing gradient problem while preserving gradient stability, which in turn enables more efficient model training.

To further assess the contribution of different attention mechanisms, another ablation study was conducted. The results, summarized in Table 3, indicate that replacing GAM with alternative attention mechanisms (CBAM, SE, ECA, SK) led to noticeable decreases in model performance, particularly in the extraction of critical fault features. GAM achieved the highest accuracy of 97.44% with a loss of 0.0740, significantly outperforming the other mechanisms. This strongly suggests that GAM offers the best balance between local and global feature extraction, leading to improved fault detection accuracy.

To further validate the superiority of the SSG-Net model, its accuracy performance was compared with that of other conventional models in the scroll compressor fault diagnosis task. The results indicate that SSG-Net significantly outperforms models such as ResNet, GAMCNN, and Swin Transformer. Specifically, the data in Table 4 show that SSG-Net achieves an accuracy of 97.44%, which represents a 32.40% improvement compared to ResNet’s 65.04%. Moreover, compared with GAMCNN and Swin Transformer, the accuracy of SSG-Net improved by 1.82% and 2.99%, respectively. Additionally, the dual-channel model Swin Transformer-GAMCNN achieves an accuracy of 96.06%, and SSG-Net still improves on this by 1.38%. In summary, the accuracy of SSG-Net in fault diagnosis tasks is significantly enhanced, demonstrating its great potential in practical applications.

To further validate the experimental results, the detection outcomes were visualized using the confusion matrix and T-SNE, while violin plots were employed to reveal the correlation between samples of the same type after classification. The confusion matrix, a commonly used graphical representation in data analysis, illustrates the relationship between the classifier’s predicted results and the actual labels in a grid format. Specifically, the rows and columns of the confusion matrix represent the actual and predicted categories, respectively. The value of each cell indicates the number or percentage of samples for which the model predicts the actual label as the corresponding label. Additionally, shades of color are utilized to indicate the number or percentage of samples for different combinations of categories, making the information more intuitive and understandable.

**Figure 9:**
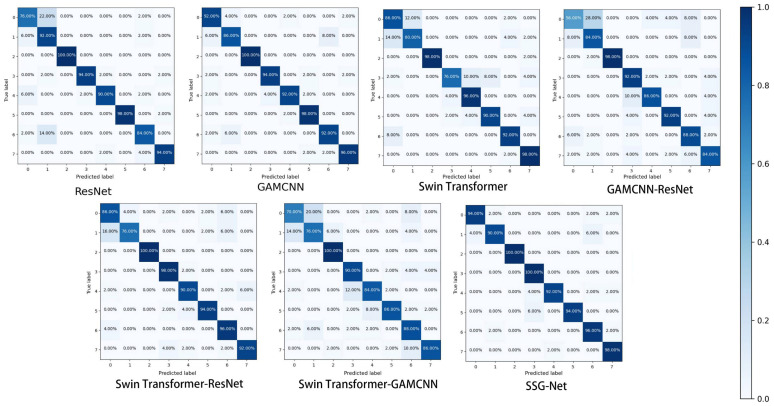
Confusion matrix.

Figure 9 illustrates the confusion matrix of the seven models for scroll compressor fault diagnosis, demonstrating their performance in terms of prediction accuracy across the categories. ResNet exhibits higher accuracy in categories 0 and 6, with 88% and 84%, respectively, but shows lower accuracy in categories 1, 3, and 4, with 72%, 52%, and 34%, respectively. The GAMCNN-ResNet model achieves 100% prediction accuracy in categories 2 and 5, with accuracy ranging between 72% and 96% in categories 0, 1, 3, 4, and 6. Similarly, Swin Transformer-ResNet attains 100% prediction accuracy in categories 2 and 5, with accuracy ranging between 84% and 96% for the remaining categories. Overall, SSG-Net demonstrates the best performance across all categories.

In a violin plot, each violin represents a labeled data distribution. A violin plot is a combination of a box-and-line plot and a density plot, showing the shape, center, and dispersion of the data distribution. The white dot in the center represents the median, the black box represents the interquartile range, and the width of the violin indicates the density of the distribution of the data points, with wider violins indicating a higher concentration of data points around that value.

**Figure 10:**
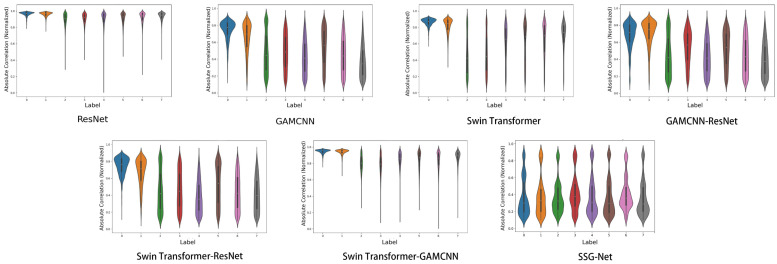
Sample correlation violin chart.

As illustrated in Figure 10, while the SSG-Net model may not achieve the same level of consistency and stability as the Swin Transformer-GAMCNN and GAMCNN-ResNet models, it excels in processing time–frequency diagram data. This is largely due to the model’s flexibility and adaptability, which enable it to capture subtle variations and complex patterns within time-frequency data. These capabilities are particularly advantageous when dealing with complex and highly heterogeneous datasets or in application scenarios that demand high labeling accuracy. Conversely, the Swin Transformer-GAMCNN and GAMCNN-ResNet models demonstrate more consistent and stable performance across all labels, making them better suited for tasks requiring high reliability and uniformity. The ResNet and Swin Transformer-ResNet models also exhibit balanced performance, albeit with some variability. On the other hand, the Swin Transformer and GAMCNN models perform well on specific labels but show greater overall variability, resulting in lower stability compared to the previously mentioned models. Overall, the SSG-Net model presents a more concentrated and stable distribution of sample correlations across all labels, characterized by lower variance and good symmetry. This suggests that the SSG-Net model not only maintains high accuracy but also provides improved prediction consistency and generalization abilities, effectively compensating for the limitations of other models in managing complex and diverse data.

**Figure 11:**
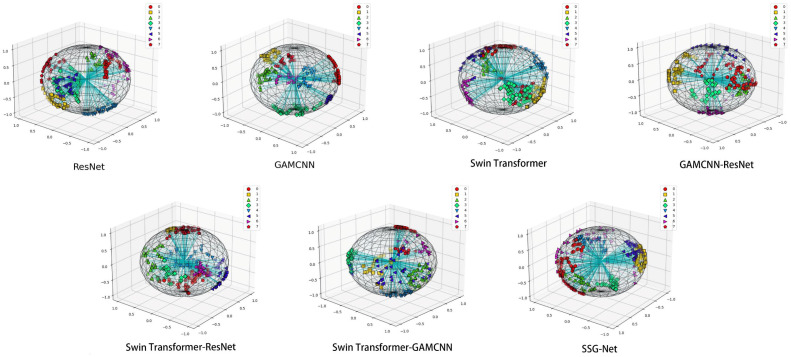
T-SNE visualization.

As shown in Figure 11, the T-SNE plot demonstrates the distribution of the SSG-Net model for different categories of samples after detection. Each color represents a different category, and most categories form clear and concentrated clusters, reflecting the similarity between samples and the differences between categories. Similar categories are clustered together with clear spacing between different categories, indicating that the model distinguishes between different categories well in most cases. However, a slight overlap between a few categories is observed, likely due to the similarity of the samples’ features in the high-dimensional space, leading to some confusion between categories.

When compared to the other six models, SSG-Net performs the best. ResNet and the GAMCNN model have relatively poor classification effects, with more crossover between data points. The Swin Transformer model, GAMCNN-ResNet model, Swin Transformer-ResNet, and Swin Transformer-GAMCNN show better classification to varying degrees, with a more even distribution of data points and a clearer demarcation between categories. Overall, SSG-Net performs best in the diagnostic tasks of different types of faults, demonstrating its strong potential in practical applications.

### 4.2. CWRU Dataset

**Figure 12:**
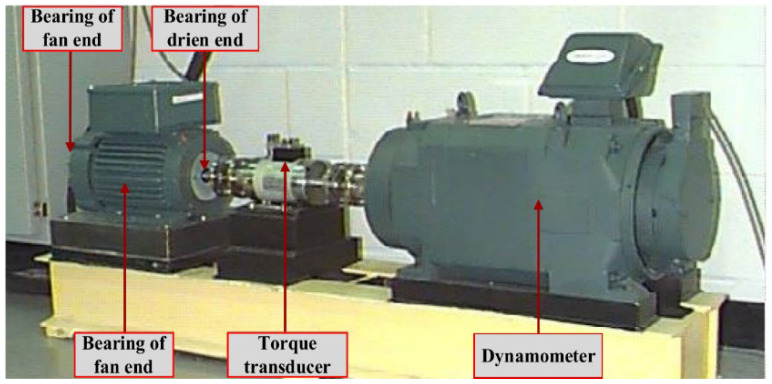
CWRU test bed.

In this experiment, the CWRU dataset was used to validate the fault diagnosis model. The equipment diagram of the CWRU testbed is shown in Figure 12. The vibration data from the CWRU dataset were collected by accelerometers at a sampling frequency of 12 kHz. Nine fault classes and one normal class from the CWRU dataset were selected for this experiment. These classes include inner ring, outer ring, and rolling body faults with fault diameters of 0.007, 0.014, and 0.021 inches. Each class has 2250 samples, resulting in a total of 22,500 training samples. The training and test sets for each class were divided in the ratio of 7:2:1, and the dataset classification is shown in Table 5.

(1) Data Processing: The one-dimensional signal was converted into a two-dimensional STFT time-frequency map, and the processing method for dataset B was identical to that of dataset A. The dataset was built after data enhancement using the CWRU-transformed time-frequency maps, enabling the model to better understand and analyze the frequency and time-domain characteristics of the signals, thereby providing rich information for subsequent detection.
(2) Comparison Experiments: In this study, several widely used deep learning models, including ResNet and multi-channel models, were compared in the field of image classification. Although the ablation experiments for selecting the most appropriate activation function were conducted using a previous dataset, the Hardswish activation function was ultimately chosen for the SSG Net model due to its demonstrated superior performance. Both accuracy and loss were consistently used as evaluation metrics throughout the comparison and validation processes.
(3) Experimental Results and Analysis: The generalization ability of the proposed method was verified. Furthermore, the proposed model demonstrated strong generalization and feature extraction abilities. To obtain more reliable results, each model was trained ten times, and the average values were subsequently calculated. The tables and figures indicate that the proposed method achieves good accuracy on the CWRU dataset compared to other models.

As shown in Table 6, there is a significant difference in the performance of different models in the fault diagnosis task when using the CWRU dataset. SSG-Net achieves an accuracy of 99.78%, significantly outperforming the other models. Specifically, SSG-Net improves the accuracy by 12.28 percentage points compared to ResNet (87.50%), 8.60 percentage points compared to GAMCNN (91.18%), 8.26 percentage points compared to Swin Transformer (91.52%), and 2.24 percentage points compared to the combined model Swin Transformer-GAMCNN (97.54%). Compared to Swin Transformer-ResNet (98.45%), SSG-Net shows an improvement of 1.33 percentage points, and compared to GAMCNN-ResNet (99.33%), it shows an improvement of 0.45 percentage points. These results demonstrate that SSG-Net exhibits excellent performance in fault diagnosis tasks, highlighting the versatility and applicability of the model.

**Figure 13:**
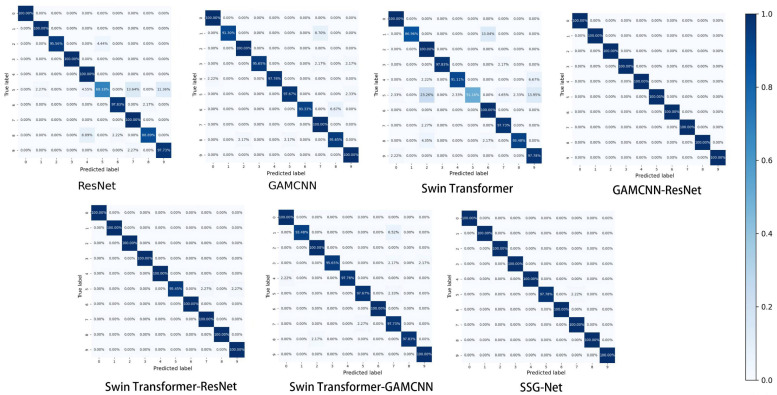
Confusion matrix.

To compare the diagnostic effectiveness of the models on different types of faults, Figure 13 presents the confusion matrix of detection results for the seven models on the CWRU dataset. ResNet achieved high prediction accuracies of 88.00% and 84.00% in categories 0 and 6, respectively, but performed poorly in categories 1, 3, and 4. In contrast, the overall performance of the GAMCNN model is more balanced, showing no significant weaknesses. Similarly, the Swin Transformer model exhibits consistent and stable prediction accuracies across all categories. Both the GAMCNN-ResNet model and Swin Transformer-ResNet achieved 100.00% prediction accuracies in categories 2 and 5, with accuracies in the other categories ranging from 72.00% to 96.00%. Notably, SSG-Net performed the best overall, demonstrating the strongest potential for application in scroll compressor fault diagnosis tasks.

**Figure 14:**
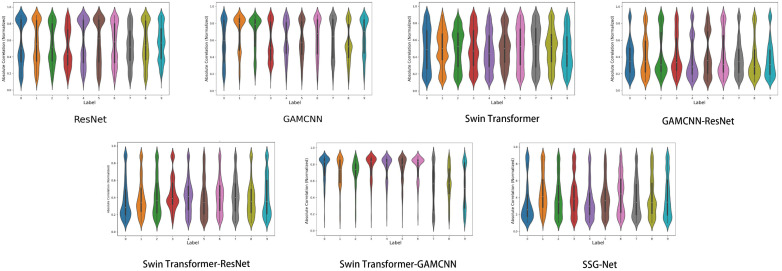
Sample correlation violin chart.

As shown in Figure 14, although SSG-Net may not be as consistent and stable as the Swin Transformer-GAMCNN and GAMCNN-ResNet models, it performs well in processing time-frequency graph data. The flexibility and adaptability of SSG-Net enable it to capture subtle differences and complex patterns in time-frequency graph data, especially when processing complex and highly heterogeneous datasets or in application scenarios where specific labels are particularly demanding. In contrast, the Swin Transformer-GAMCNN and GAMCNN-ResNet models perform more consistently and stably across all labels, making them suitable for tasks requiring high reliability and consistency. ResNet and Swin Transformer-ResNet also exhibit balanced performance but with some variability. The Swin Transformer and GAMCNN models perform well on some labels, but their overall variability and lack of stability are greater than those of the aforementioned models. In conclusion, although SSG-Net is slightly less consistent and stable, its excellent performance and flexible adaptability in fault diagnosis make it an ideal choice for processing complex time-frequency graph data.

**Figure 15:**
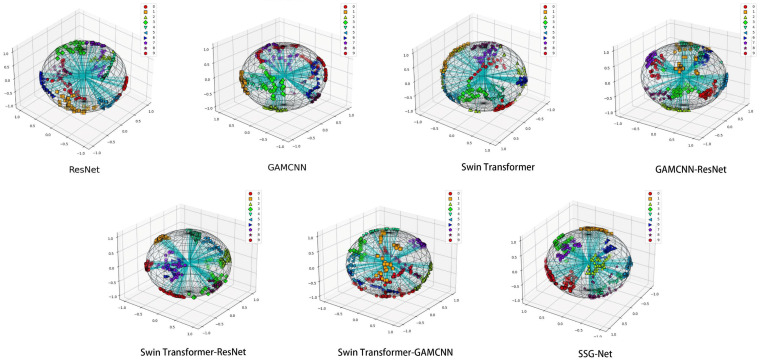
T-SNE visualization.

As shown in Figure 15, the T-SNE visualization demonstrates that SSG-Net achieves the best classification effect, with the most uniform distribution and significant aggregation of data points in each category. In contrast, ResNet and the GAMCNN model exhibit relatively poor classification effects, with more crossings between data points. Other models, such as the Swin Transformer model, GAMCNN-ResNet model, Swin Transformer-ResNet, and Swin Transformer-GAMCNN, show better classification effects to varying degrees. Overall, SSG-Net performs the best in diagnostic tasks for different types of faults.

## 5. Conclusions

To overcome the limitations of traditional data-driven and model-driven methods in compressor fault detection, a novel deep feature learning framework, SSG-Net, has been introduced. By incorporating STFT, Swin Transformer, Shallow ResNet, and the GAM global attention mechanism, this framework effectively captures both time-frequency and local spatial features, significantly enhancing fault signal recognition. Moreover, the Swin Transformer’s multi-scale feature extraction, when combined with the GAM mechanism’s global attention, substantially improves the model’s robustness and accelerates its convergence, enabling highly efficient compressor fault detection. Experimental results demonstrate that SSG-Net consistently surpasses existing models on both the scroll compressor dataset and a widely used public dataset, especially in terms of recognition accuracy and convergence speed. Consequently, these strengths establish SSG-Net as a highly competitive approach for practical applications.

In future research, the method’s robust feature extraction and generalization capabilities can be further leveraged to improve fault diagnosis across diverse scenarios, such as varying operating conditions, small-sample learning, and life prediction. However, the current approach is constrained by its limitation to identifying only the fault categories present in the training set, with a reduced ability to detect or manage unknown faults. To address these shortcomings and better align with the complexities of real-world applications, future studies should focus on testing the model under more challenging conditions, including noisy environments, cross-domain data, and imbalanced datasets.

Furthermore, to mitigate the challenges associated with small-sample learning, transfer learning and few-shot learning techniques should be explored. These approaches could enable the model to adapt swiftly to new working conditions or fault types using minimal data. Additionally, Generative Adversarial Networks (GANs) could be utilized to generate synthetic fault samples, enriching the training dataset and further enhancing the model’s generalization capabilities. By implementing these advancements, the method is expected to provide more comprehensive and reliable fault diagnosis, offering stronger support for equipment maintenance and fault management in complex and dynamic industrial environments.

## Figures and Tables

**Table 1:** Compressor dataset.

Fault Type	Label	Training Samples	Test Samples	Validation Samples
Normal	0	1575	450	225
BLF	1	1575	450	225
OVF	2	1575	450	225
IVF	3	1575	450	225
CWF	4	1575	450	225
RIF	5	1575	450	225
SDF	6	1575	450	225
MALF	7	1575	450	225

**Table 2:** Performance of SSG-Net with different activation functions.

Model	Accuracy/%	Loss
SSG-Net + SELU	94.28	0.1655
SSG-Net + ReLU	96.06	0.1159
SSG-Net + ELU	93.67	0.1863
SSG-Net + Hardswish	97.44	0.0740
SSG-Net + LackyRELU	91.82	0.1983

**Table 3:** Performance comparison of different attention mechanisms.

Attention Mechanism	Accuracy/%	Loss
GAM	97.44	0.0740
CBAM	92.97	0.2507
SE	94.34	0.1688
ECA	93.58	0.1864
SK	93.39	0.1915

**Table 4:** Results of the multi-model comparison.

Model	Accuracy/%	Loss
ResNet	65.04	1.0024
GAMCNN	95.62	0.1219
Swin Transformer	94.45	0.1531
Swin Transformer-GAMCNN	96.06	0.1159
Swin Transformer-ResNet	92.63	0.2141
GAMCNN-ResNet	87.75	0.3586
SSG-Net	97.44	0.0740

**Table 5:** Classification of the CWRU dataset.

Fault Type	Fault Diameter	Label	Training Samples	Test Samples	Validation Samples
Normal	0.007	0	1575	450	225
inner		1	1575	450	225
Ball		2	1575	450	225
outer		3	1575	450	225
inner	0.014	4	1575	450	225
Ball		5	1575	450	225
outer		6	1575	450	225
inner	0.021	7	1575	450	225
Ball		8	1575	450	225
outer		9	1575	450	225

**Table 6:** Comparison of multi-model results.

Model	Accuracy/%	Loss
ResNet	87.50	0.220
GAM-CNN	91.18	0.048
Swin Transformer	91.52	0.164
Swin Transformer-GAMCNN	97.54	0.0819
Swin Transformer-ResNet	98.45	0.106
GAMCNN-ResNet	99.33	0.071
SSG-Net	99.78	0.408

## Data Availability

The raw data required to reproduce these findings cannot be shared at this time as the data also form part of an ongoing study.

## References

[1] Hareland M., Hoel A., Jonsson S., Liang D. Selection of Flapper Valve Steel for High Efficient Compressor. Proceedings of the International Compressor Engineering Conference.

[2] Dufour D., Le Noc L., Tremblay B., Tremblay M.N., Généreux F., Terroux M., Vachon C., Wheatley M.J., Johnston J.M., Wotton M. (2021). A Bi-Spectral Microbolometer Sensor for Wildfire Measurement. Sensors.

[3] Kang S.M., Yang E.S., Shin J.U., Park J.H., Lee S.D., Ha J.H., Son Y.B., Lee B.C. Development of High Speed Inverter Rotary Compressor for the Air-conditioning System. Proceedings of the 9th International Conference on Compressors and their Systems.

[4] Tian Z., Gu B. (2019). Analyses of an integrated thermal management system for electric vehicles. Int. J. Energy Res..

[5] Choi Y.U., Kim M.S., Kim G.T., Kim M., Kim M.S. (2017). Analyse de la performance d’un système de pompe à chaleur à injection de vapeur pour les véhicules électriques devant démarrer sous températures froides. Int. J. Refrig..

[6] Li K., Ma J., Cao J., Zhang B., Dou B., Liu N., Zhang H., Su L., Zhou X., Tu R. (2023). The influences of the oil circulation ratio on the performance of a vapor injection scroll compressor in heat pump air conditioning system intended for electrical vehicles. Int. J. Refrig..

[7] Peng M., Peng X., Wang D., Liu X., Yang Y., Wang G., Chen B. (2022). Investigation of the unsteady characteristic in a scroll compressor of a heat pump system for electric vehicles. J. Therm. Anal. Calorim..

[8] Fu W., Shao K., Tan J., Wang K. (2020). Fault diagnosis forrolling bearings based on composite multiscale fine-sorteddispersion entropy and SVM with hybrid mutation SCA-HHO algorithm optimization. IEEE Access.

[9] Hsiao T., Weng M.A. (2012). hierarchical multiple-model approach for detection and isolation of robotic actuator faults Robot. Robot. Auton. Syst..

[10] Hu X., Cao Y., Tang T., Sun Y. (2022). Data-driven technology of fault diagnosis in railway point machines: Review and challenges. Transp. Saf. Environ..

[11] Gao Z., Cecati C., Ding S. (2015). A survey of fault diagnosis and fault-tolerant techniques Part I: Fault diagnosis with model-based and signal-based approaches. IEEE Trans. Ind. Electron..

[12] Chen X., He K. Exploring simple Siamese representation learning. Proceedings of the IEEE/CVF Conference on Computer Vision and Pattern Recognition.

[13] Fu W., Wang K., Zhang C., Tan J. (2019). A hybrid approach for measuring the vibrational trend of hydroelectric unit with enhanced multi-scale chaotic series analysis and optimized least squares support vector machine. Trans. Inst. Meas. Control..

[14] Cao Y., Song D., Hu X., Sun Y. (2023). Fault diagnosis of railway point machine based on improved time-domain multiscale dispersion entropy and support vector machine. Acta Electron. Sin..

[15] Hua L., Zhang C., Peng T., Ji C. (2022). Integrated framework of extreme learning machine (ELM) based on improved atom search optimization for short-term wind speed prediction Energy Convers. Energy Convers. Manag..

[16] Gumaei A., Hassan M.M., Hassan R., Alelaiwi A., Fortino G. (2019). A Hybrid Feature Extraction Method with Regularized Extreme Learning Machine for Brain Tumor Classification. IEEE Access.

[17] Lu J., Qian W., Li S., Cui R. (2021). Enhanced K-nearest neighbor for intelligent fault diagnosis of rotating machinery. Appl. Sci..

[18] Cunningham P., Delany S.J. (2021). K-Nearest Neighbour Classifiers—A Tutorial. ACM Comput. Surv. (CSUR).

[19] Rastinn, Jahromim Z. (2021). Taherim A generalized weighted distance k-Nearest Neighbor for multi-label problems. Pattern Recognit..

[20] Fu Q., Jing B., He P., Si S., Wang Y. (2018). Fault Feature Selection and Diagnosis of Rolling Bearings Based on EEMD and Optimized Elman AdaBoost Algorithm. IEEE Sens. J..

[21] Sun Y., Li S., Wang X. (2021). Bearing fault diagnosis based on EMD and improved Chebyshev distance in SDP image. Measurement.

[22] Chen F., Tang B., Song T., Li L. (2014). Multi-fault diagnosis study on roller bearing based on multi-kernel support vector machine with chaotic particle swarm optimization. Measurement.

[23] Ma C., Gu X., Wang Y. (2009). Fault diagnosis of power electronic system based on fault gradation and neural network group. Neurocomputing.

[24] Liu R., Yang B., Zio E., Chen X. (2018). Artificial intelligence for fault diagnosis of rotating machinery: A review. Mech. Syst. Signal Process..

[25] Dong Y., Jiang H., Wu Z., Yang Q., Liu Y. (2023). Digital twin-assisted multiscale residual-self-attention feature fusion network for hypersonic flight vehicle fault diagnosis. Reliab. Eng. Syst. Saf..

[26] Chen X., Hu X., Wen T., Cao Y. (2023). Vibration Signal-Based Fault Diagnosis of Railway Point Machines via Double-Scale CNN. Chin. J. Electron..

[27] Dong Y., Jiang H., Liu Y., Yi Z. (2024). Global wavelet-integrated residual frequency attention regularized network for hypersonic flight vehicle fault diagnosis with imbalanced data. Eng. Appl. Artif. Intell..

[28] Zhao X., Yao J., Deng W., Ding P., Ding Y., Jia M., Liu Z. (2022). Intelligent fault diagnosis of gearbox under variable working conditions with adaptive intraclass and interclass convolutional neural network. IEEE Trans. Neural Netw. Learn. Syst..

[29] Liu S., Chen J., He S., Shi Z., Zhou Z. (2023). Few-shot learning under domain shift: Attentional contrastive calibrated transformer of time series for fault diagnosis under sharp speed variation. Mech. Syst. Signal Process..

[30] He Z., Shen C., Chen B., Shi J., Huang W., Zhu Z., Wang D. (2024). A new feature boosting based continual learning method for bearing fault diagnosis with incremental fault types. Adv. Eng. Inform..

[31] Seimert M., Gühmann C. (2017). Vibration based diagnostic of cracks in hybrid ball bearings. Measurement.

[32] (2021). Yang Z, Gjorgjevikj D, Long J, Zi Y, Zhang S, Li C Sparse autoencoder-based multi-head deep neural networks for machinery fault diagnostics with detection of novelties. Chin. J. Mech. Eng..

[33] Lei Y., He Z., Zi Y. (2008). A new approach to intelligent fault diagnosis of rotating machinery. Expert Syst. Appl..

[34] Liu T., Li L., Noman K., Li Y. (2024). Local maximum instantaneous extraction transform based on extended autocorrelation function for bearing fault diagnosis. Adv. Eng. Inform..

[35] Liu S., Yin J., Hao M., Liang P., Zhang Y., Ai C., Jiang W. (2024). Fault diagnosis study of hydraulic pump based on improved symplectic geometry reconstruction data enhancement method. Adv. Eng. Inform..

[36] Deng L., Li W., Zhang W. (2023). Intelligent prediction of rolling bearing remaining useful life based on probabilistic DeepAR-Transformer model. Meas. Sci. Technol..

[37] Jin T., Yan C., Chen C., Yang Z., Tian H., Guo J. (2021). New domain adaptation method in shallow and deep layers of the CNN for bearing fault diagnosis under different working conditions. Int. J. Adv. Manuf. Technol..

[38] Taylor L., Nitschke G. Improving deep learning with generic data augmentation. Proceedings of the 2018 IEEE Symposium Series on Computational Intelligence.

[39] Fernandez A., Garcia S., Herrera F., Chawla N.V. (2018). SMOTE for Learning from Imbalanced Data: Progress and Challenges, Marking the 15-year Anniversary. J. Artif. Intell. Res..

[40] He H., Bai Y., Garcia E.A., Li S. ADASYN: Adaptive synthetic sampling approach for imbalanced learning. Proceedings of the IEEE International Joint Conference on Neural Networks.

[41] Jiao J., Li H., Zhang T., Lin J. (2022). Source-free adaptation diagnosis for rotating machinery. IEEE Trans. Ind. Inform..

[42] Hu T., Tang T., Lin R., Chen M., Han S., Wu J. (2020). A simple data augmentation algorithm and a self-adaptive convolutional architecture for few-shot fault diagnosis under different working conditions. Measurement.

[43] Yang B., Lei Y., Jia F., Li N., Du Z. (2019). A Polynomial kernel induced distance metric to improve deep transfer learning for fault diagnosis of machines. IEEE Trans. Ind. Electron..

[44] Goodfellow I., Pouget-Abadie J., Mirza M., Xu B., Warde-Farley D., Ozair S., Courville A., Bengio Y. (2020). Generative adversarial networks. Commun. ACM.

[45] Jiao J., Li H., Lin J. (2022). Self-training reinforced adversarial adaptation for machine fault diagnosis. IEEE Trans. Ind. Electron..

[46] Zhao M., Zhong S., Fu X., Tang B., Pecht M. (2019). Deep residual shrinkage networks for fault diagnosis. IEEE Trans. Ind. Inform..

[47] Zhang T., Chen J., Li F., Pan T., He S. (2020). A small sample focused intelligent fault diagnosis scheme of machines via multimodules learning with gradient penalized generative adversarial networks. IEEE Trans. Ind. Electron..

[48] Gao X., Deng F., Yue X. (2019). Data augmentation in fault diagnosis based on the Wasserstein generative adversarial network with gradient penalty. Neurocomputing.

[49] Xiao Y., Shao H., Wang J., Yan S., Liu B. (2024). Bayesian variational transformer: A generalizable model for rotating machinery fault diagnosis. Mech. Syst. Signal Process..

[50] Fang H., Deng J., Chen D., Jiang W., Shao S., Tang M., Liu J. (2023). You can get smaller: A lightweight self-activation convolution unit modified by transformer for fault diagnosis. Adv. Eng. Inform..

[51] Liang P., Yu Z., Wang B., Xu X., Tian J. (2023). Fault transfer diagnosis of rolling bearings across multiple working conditions via subdomain adaptation and improved vision transformer network. Adv. Eng. Inform..

[52] Tang J., Zheng G., Wei C., Huang W., Ding X. (2022). Signal-transformer: A robust and interpretable method for rotating machinery intelligent fault diagnosis under variable operating conditions. IEEE Trans. Instrum. Meas..

[53] Ding Y., Jia M., Miao Q., Cao Y. (2022). A novel time–frequency Transformer based on self–attention mechanism and its application in fault diagnosis of rolling bearings. Mech. Syst. Signal Process..

[54] Zhao D., Cai W., Cui L. (2024). Adaptive thresholding and coordinate attention-based tree-inspired network for aero-engine bearing health monitoring under strong noise. Adv. Eng. Inform..

[55] Huang Y., Shi P., He H., He H., Zhao B. (2023). Senet: Spatial information enhancement for semantic segmentation neural networks. Vis. Comput..

[56] Wang S.-H., Fernandes S.L., Zhu Z., Zhang Y.-D. (2021). AVNC: Attention-based VGG-style network for COVID-19 diagnosis by CBAM. IEEE Sens. J..

[57] Park J., Woo S., Lee J.-Y., Kweon I.S. (2018). Bam: Bottleneck attention module. arXiv.

[58] Liu Y., Shao Z., Hoffmann N. (2021). Global attention mechanism: Retain information to enhance channel-spatial interactions. arXiv.

[59] He K., Zhang X., Ren S., Sun J. Deep residual learning for image recognition. Proceedings of the IEEE Conference on Computer Vision and Pattern Recognition.

